# Consistency in patient-reported outcome measures after total knee arthroplasty using patient-specific instrumentation: a 5-year follow-up of 200 consecutive cases

**DOI:** 10.1007/s00167-017-4800-7

**Published:** 2017-11-17

**Authors:** Daphne A. L. Schoenmakers, Martijn G. M. Schotanus, Bert Boonen, Nanne P. Kort

**Affiliations:** Department of Orthopaedic Surgery, Zuyderland Medical Center, Dr. H. van der Hoffplein 1, 6162 BG Sittard-Geleen, The Netherlands

**Keywords:** Total knee arthroplasty, Total knee replacement, Patient-specific instrumentation, Implant survival, Patient reported outcome measures

## Abstract

**Purpose:**

The purpose of this study was to evaluate the 5-year follow-up results of the first 200 total knee arthroplasties (TKA) performed by one high-volume surgeon, using patient-specific information (PSI). To date, there has been no other research into the mid-term follow-up of TKA performed using PSI.

**Materials and methods:**

A total of 184 consecutive patients (200 TKA) were evaluated. Outcome measures included implant survival rate, adverse events, and the following patient-reported outcome measures (PROMs); Western Ontario and McMaster Universities Osteoarthritis Index (WOMAC), Oxford Knee Score (OKS), Pain Visual Analogue Score (VAS) and EuroQol-5D Score (EQ-5D).

**Results:**

Revision surgery was performed for late secondary prosthetic joint infection (*n* = 1, total revision), aseptic loosening (*n* = 1, tibial component revision), instability (*n* = 1, isolated polyethylene insert exchange), and polyethylene insert breakage (*n* = 1, isolated polyethylene insert exchange). Other adverse events were as follows: debridement, antibiotics and implant retention for early prosthetic joint infection (*n* = 1), surgical debridement for haemarthrosis (*n* = 1), superficial wound infection (*n* = 2), thromboembolic events (*n* = 2), compartment syndrome (*n* = 1), and nerve injury (*n* = 2). All median outcome scores for patient reported outcome measures at 5 years improved significantly compared with the preoperative values (*p* ≤ 0.05). Median outcome scores were not significantly different between 1- and 5-year moments of follow-up, except for a significant decrease of EQ-VAS (*p* ≤ 0.05) between these two follow-up moments.

**Conclusion:**

PROMs are consistent for 5-year follow-up of TKA using PSI. After 5 years of follow-up, revision surgery for any reason occurred in four patients (2%).

**Level of Evidence:**

III.

## Introduction

Positioning of knee prosthesis components and lower limb alignment after total knee arthroplasty (TKA) are important factors influencing implant survival and clinical results [17, 19, 25]. Surgical techniques have evolved over time, and now there are several methods used that assist in obtaining the desired alignment of TKA. One of these methods includes patient-specific instrumentation (PSI). PSI uses guides based on a preoperative MRI- or CT-scan of the patient’s leg. This technology has the potential to increase cost-effectiveness due to the reduction in surgical time and the need for fewer surgical trays [24].

Many previous studies have compared alignment obtained with PSI to standard instrumentation, with mixed results [1, 3–5, 9, 10, 13, 15, 21]. Research on the use of PSI shows a reduction in surgical time [4, 5, 8, 10, 21, 24], blood loss [4, 5, 9, 24], and hospital stay [9, 21] in comparison to conventional instrumentation. Others did not find significant differences in surgical time [1], blood loss [1, 8, 21], or hospital stay [1, 4, 8, 24].

Fewer studies focused on short-term functional follow-up results, which shows similar good outcomes when compared to conventional instrumentation [1, 6, 20]. However, no data exists with regard to longer follow-up results of TKA performed using PSI. In continuation of the previous study by Boonen et al. [6], this study presents the 5-year follow-up results of the first 200 consecutive cases operated on by one single high-volume surgeon. The focus of this study is on implant survival rate, adverse events, and on patient reported outcome measures (PROMs). The authors hypothesize that results of TKA performed using PSI after 5 years of follow-up show similar good outcomes compared to earlier follow-up.

## Materials and methods

Data were collected from the first 184 patients, on whom 200 TKA were performed using PSI. The data consisted of patient records of their preoperative appointment and routine 1-, 2-, and 5-year follow-up appointments.

### Cohort and surgical technique

The Signature™ system (Zimmer-Biomet Inc., Warsaw, IN) was used in this cohort. The patients underwent surgery between July 2009 and March 2011. Inclusion and exclusion criteria, as well as surgical techniques and perioperative management are described in previous reports [5, 6].

Baseline characteristics are listed in Table 1.

**Table 1:** Baseline characteristics

Characteristic	Median interquartile range (IQR), or absolute numbers (%)
Females, *n* (%)	106 (57.6%)
Median age at surgery date, years (IQR)	68.1 (60.7–74.6)
Bilateral TKA cases, *n* (%)	16 (8.7%)
Median follow-up, years (IQR)	5.5 (5.2–5.9)

### Outcome measurements

Implant survival rate was defined as revision surgery for any reason. All revision surgeries and adverse events were recorded during the 5-year follow-up.

Preoperatively, patients completed the following questionnaires: the Western Ontario and McMaster Osteoarthritis Index (WOMAC; scored from 0 to 100, 0 being the worst outcome and 100 being the best possible outcome) [27], the Oxford Knee Score (OKS; scored from 12 to 60, with 12 being the worst outcome and 60 being the best possible outcome) [12], the Pain Visual Analogue Score (VAS; scored from 0 to 10, 0 representing no pain and 10 representing the worst imaginable pain) and the EuroQol (The EQ-5D-3L) including the EQ-Index (scored from 1 to 3, 1 represents perfect health and no disabilities) and EQ-Visual Analogue Scale (EQ-VAS; records the respondent’s own assessment of their health status on a vertical VAS where the scores are anchored on 100 equal to ‘Best imaginable health state’ and 0 equal to ‘Worst imaginable health state’) [7]. The health states, defined by the EQ-5D-3L, can be converted to a single index value using the calculator provided by The EuroQol Group.

Scores on the questionnaires were compared between the different follow-up visits. The same set of questionnaires was completed by patients themselves right before their appointments preoperatively, and 1, 2, and 5 years postoperatively.

At the time of 5-year follow-up 11 patients (12 TKA, 6%) were deceased of causes unrelated to TKA. Of the patients, 116 (128 TKA, 64%) attended their 5-year follow-up appointment, while 57 (60 TKA, 30%) cancelled their follow-up appointment, and three (3 TKA, 1.5%) did not complete the questionnaires. The aforementioned questionnaires were sent out by post to these 60 patients (63 TKA, 31.5%); 54 patients returned their questionnaires (57 TKA, 28.5%), two (2 TKA, 1%) were not able to participate due to Alzheimer’s disease, one (1 TKA, 0.5%) declined to participate, and three others (3 TKA, 1.5%) did not respond, so additional information could not be obtained. These six patients (6 TKA, 3%) were considered lost to follow-up.

**Fig. 1:**
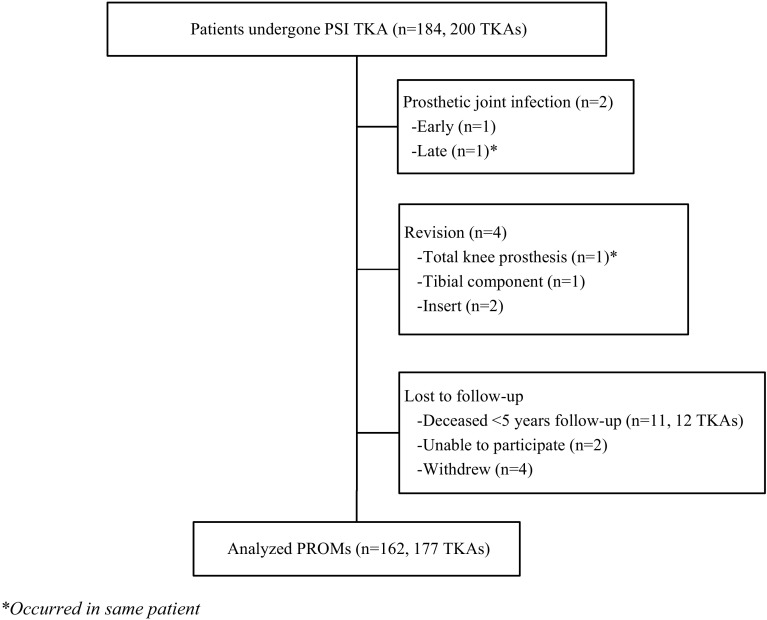
Diagram of the number of patients enrolled in the study, patients with revision or prosthetic joint infection, amount of patients lost to follow-up, and analysed PROMs. *Occurred in same patient

Five patients (5 TKA, 2.5%) were excluded from overall analysis of the questionnaires because of revision surgery or prosthetic joint infection. PROMs were evaluated from 162 patients (177 TKA, 88.5%), with a median follow-up of 5.5-years (IQR 5.2–5.9 years) (Fig. 1).

### Institutional review board approval

Institutional review board (METC Z, Heerlen, the Netherlands) approval was obtained for the study (trial number 12-N-139).

### Statistical analysis

All statistical analyses were performed using SPSS software version 20.0 (SPSS Inc., Chicago, Illinois). Descriptive statistics were used for baseline characteristics. The Shapiro–Wilk test showed that data were not normally distributed. Therefore, Wilcoxon signed ranks tests were performed on significant interactions. A threshold for all statistical comparisons of *p* value ≤ 0.05 was considered to be statistically significant. Data are presented as median with interquartile ranges (IQR) or with frequencies.

## Results

After 5 years of follow-up, four patients (4 TKA, 2%) had undergone revision surgery (Fig. 1). One patient required total revision for a secondary haematogenic prosthetic joint infection associated with a colon ascendens tumour (26.4 months after TKA). Prior to this two-step revision, the patient underwent debridement, antibiotics, and implant retention in another medical centre. Furthermore, revision of the tibial component was required in one patient for aseptic loosening (25.7 months after TKA). Isolated polyethylene insert exchange with retention of total knee prosthesis occurred in two patients due to instability of collateral ligaments or breakage of the polyethylene insert after trauma (30.0 and 44.4 months after TKA, respectively).

One patient received debridement, antibiotics, and implant retention for early prosthetic joint infection (16 days after TKA). Surgical (arthroscopic) debridement was done in one patient to alleviate pain due to haemarthrosis (44 days after placement of TKA). One patient required fasciotomy due to compartment syndrome 5 days after surgery. Two patients received oral antibiotics for superficial wound infections. Other complications were pulmonary embolism 6 days after surgery (*n* = 1), minor stroke 9 days after surgery (*n* = 1), femoral nerve lesion after femoral nerve block anaesthesia (*n* = 1), and temporary tibial nerve neuropraxia (*n* = 1).

PROMs measured preoperatively and at the follow-up moments are shown in Table 2. After 5 years of follow-up, all median outcome scores for PROMs improved significantly from preoperative values (*p* ≤ 0.05). No significant differences were observed between postoperative scores 1, 2 or 5 years after the operation, except for a significant decrease of EQ-VAS from 1- to 5-year follow-up (*p* = 0.002).

**Table 2:** Results at the different follow-up visits presented as median scores and interquartile range

	Preoperative	1-year postoperative	2-year postoperative	5-year postoperative
WOMAC	57 (39–79)	91 (72–97)*	90 (72–98)*	90 (68–98)*
OKS	21 (16–26)	41 (36–45)*	41 (33–45)*	42 (33–46)*
VAS	7 (6–8)	2 (0–5)*	2 (0–5)*	1 (0–5)*
EQ-Index	0.788 (0.719–0.805)	0.874 (0.805–1.00)*	0.874 (0.805–1.00)*	0.874 (0.805–1.00)*
EQ-VAS	60 (50–80)	80 (70–90)*	80 (70–90)*	80 (60–90)*#

*Significant difference from preoperative score (*p* < 0.01)

#Significant difference from 1-year postoperative score (*p* = 0.002)

*WOMAC* Western Ontario McMaster Universities Osteoarthritis Index, *OKS* Oxford Knee Score (OKS), *VAS* Pain Visual Analogue Score (VAS), *EQ-Index and EQ-VAS* EQ-5D-3L

## Discussion

The most important finding of the present study is that 5-year follow-up results from 200 TKA using PSI show similar good outcomes in PROMs compared to the 1-year follow-up. Also, revision for any reason occurred in four patients (2%), which is well within range of the 5-year TKA survival of 93–97% as found by others [11, 16]. Sadoghi et al. [28] used worldwide arthroplasty registers to evaluate the reasons for revision. The authors found aseptic loosening (29.8%), septic loosening (14.8%), and pain (9.5%) as most common causes for revision. Instability and implant breakage accounted for 6.2 and 4.7%, respectively [28]. In the present study, we found aseptic and septic loosening to account for 50% of all revisions. Furthermore, Boonen et al. [3] found no significant different occurrence of adverse events between PSI and conventional instrumented TKA. While the sample size in the current study (*n* = 200) is too small for drawing valid conclusions on reasons for revision, the revision rates reported here are not strikingly different from the results found by the aforementioned authors.

Our data include two patients (1%) with prosthetic joint infection; which is similar to the rates reported in literature. Kurtz et al. [18] identified prosthetic joint infection incidence of 1.55% within 2 years and 0.46% between 2 and up to 10 years follow-up. Pulido et al. [23] found a similar incidence of 1.1% with a mean time to diagnosis occurring 431 days after surgery. Other studies identified an infection incidence of 1.8% up to 3.6% [22, 29].

PROMs are considered to represent the best objective measurement of the patients’ own health perception [26]. Nonetheless, PROMs remain inherently subjective, prone to an individual’s interpretation and perception of joint functioning [14, 26]. The authors of the present study received several times the feedback from patients that they found it difficult to score the PROMs. Additionally, patients described having difficulties in keeping other conditions or illnesses out of consideration that might have impaired mobility, general condition or quality of life. In the present study, PROMs measuring health-related quality of life were used next to domain-specific PROMs (e.g. pain, function, satisfaction after TKA) to provide a holistic and global approach to the TKA outcome assessment, as suggested by Hossain et al. [14]. Several studies proposed the use of performance-based measures as an addition to PROMs [2, 14]. Especially in younger patients, this may be of added value to future prospective studies.

In this study it was shown that almost all PROMs did not significantly differ between 1- and 5 years after TKA using PSI. Only the EQ-VAS (self-rated health score) decreased significantly at the 5-year follow-up compared to 1 year postoperatively. Due to aging and associated health issues, decreased self-rated health scores could be a logical consequence. Yet, the self-rated health score was still significantly better than the preoperative value. This may show that problems resulting from knee arthritis alone only partially determine the overall health score.

The strength of this study is that it is the first study that presents 5-year follow-up results of TKA performed using PSI. Furthermore, 200 consecutive patients, operated on by one single surgeon were evaluated. Therefore, the clinical relevance of this study lays in the confirmation of good mid-term results that can be expected from PSI TKA, in terms of implant survival, adverse events, and PROMs. A limitation of this study was the usage of only one PSI system, therefore these results may not be applicable to all existing PSI systems. Moreover, the present study did not directly compare results from TKA using PSI with results of conventional TKA or other surgical techniques. In addition, our study exclusively contains cases from a high-volume TKA surgeon, whereas results may not be the same for lower volume surgeons. Consequently, future research should focus on comparing PSI with other surgical techniques and PSI usage in lower volume surgeons. Furthermore, future research should assess longer follow-up results of TKA using PSI once these data are available.

## Conclusion

PROMs are consistent for 5-year follow-up of TKA using PSI. After 5 years of follow-up, revision surgery for any reason occurred in four patients (2%). Building on the findings in this study, future research should focus on the follow-up results of PSI longer than 5 years after surgery, the usage of PSI in lower volume surgeons, and the comparison with other surgical techniques for performing TKA.
